# Multispectral Chiral Quasi‐Bound States in the Continuum Enabled Microfluidics for High‐Throughput Molecular Screening and Quantification

**DOI:** 10.1002/advs.202515443

**Published:** 2025-09-28

**Authors:** Xinyue Liang, Zihan Zhao, Xiaocong Tang, Haiyue Yang, Lanju Liang, Meng Zhao, Cong Wang, Lei Wang, Xumin Ding

**Affiliations:** ^1^ Advanced Microscopy and Instrumentation Research Center School of Instrumentation Science and Engineering Harbin Institute of Technology Harbin 150080 China; ^2^ Department of Microwave Engineering Harbin Institute of Technology Harbin 150001 China; ^3^ State Key Laboratory of Advanced Inorganic Fibers and Composites School of Chemistry and Chemical Engineering Harbin Institute of Technology Harbin 150001 China; ^4^ School of Opto‐electronic Engineering Zaozhuang University Zaozhuang 277160 China; ^5^ Jiangsu Key Laboratory of Micro and Nano Heat Fluid Flow Technology and Energy Application School of Mathematics and Physics Suzhou University of Science and Technology Suzhou 215009 China

**Keywords:** chiral quasi‐bound states in the continuum, metachip, microfluidics, molecular detection

## Abstract

Traditional chiral detection methods often suffer from label dependence and limited throughput, hindering accurate profiling of trace enantiomers. Herein, a terahertz (THz) multispectral metachip‐enabled microfluidic platform is presented for label‐free, high‐throughput screening and quantitation of chiral molecules. The metachip integrates arrayed pixels engineered with chiral quasi‐bound states in the continuum (q‐BIC), generating high circular dichroism (CD) across 0.5–2.0 THz. On‐chip microfluidic integration enables multi‐dimensional CD feature extraction in aqueous environments, while the Uniform Manifold Approximation and Projection (UMAP) algorithm maps multidimensional CD features into a Two‐dimensional (2D) spectrum, simultaneously realizing conformation identification and concentration quantification (0.05–0.3 mg dL^−1^). Experimentally, 87.5% discrimination accuracy is achieved for 8 chiral biomolecules in aqueous solutions (including amino acids and dipeptides) with state‐of‐the‐art sensitivity (2.1024 THz· mg^1^ ·dL). This platform bridges the gap between high‐sensitivity chiral sensing and real‐time fluidic analysis, offering a transformative tool for trace enantiomer characterization in clinical diagnostics and drug development.

## Graphical abstract

A microfluidics‐integrated chiral quasi‐BIC metachip is developed that generates strong broadband CD from 0.5–2.0 THz in aqueous environments. A UMAP algorithm processes the resulting multidimensional CD features for simultaneous biomolecular conformation identification and concentration quantification (0.05–0.3 mg dL^−1^). This method achieves 87.5% discrimination accuracy for eight distinct chiral biomolecules with a state‐of‐the‐art sensitivity of 2.1024 THz·mg^−1^·dL.

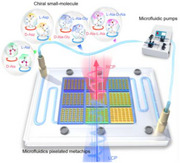

## 1 Introduction

As critical biomarkers for disease diagnosis, prognosis evaluation, drug response monitoring, pharmacodynamic studies, and personalized medicine, chiral small molecules demand the development of ultrasensitive detection technologies.^[^ ^1^ ^]^ Conventional analytical approaches, such as vibrational circular dichroism (VCD),^[^ ^2^, ^3^ ^]^ electronic circular dichroism (ECD),^[^ ^3^, ^4^ ^]^ and Raman optical activity (ROA)^[^ ^5^ ^]^ spectroscopy, face significant technical constraints due to intrinsic sensitivity limitations and operational complexity. Notably, biochemical fingerprints predominantly reside in low‐energy THz vibrational modes,^[^ ^6^, ^7^ ^]^ while chirality discrimination typically relies on infrared‐visible range circular dichroism signals with diminished characteristic fingerprint signatures. This creates an unmet need for a transformative THz chiroptical platform that simultaneously addresses both enantioselectivity and molecular identification requirements.

While traditional THz chiral sensing technologies offer fingerprint‐specific molecular identification,^[^ ^8^, ^9^ ^]^ their applicability for detecting and quantifying chiral small molecules remains compromised by low *Q*‐factors and restricted refractive index sensitivities.^[^ ^10^, ^11^, ^12^ ^]^ The development of advanced chiral detection platforms necessitates the holistic integration of high sensitivity, specificity, throughput, and miniaturization.^[^ ^13^ ^]^ 2D electromagnetic devices based on deep‐subwavelength metasurface structures have emerged as a promising paradigm to enable these competing performance metrics through precise nanoscale field confinement.^[^ ^14^, ^15^, ^16^, ^17^, ^18^, ^19^ ^]^ Particularly, q‐BIC represent optimal candidate solutions, combining theoretically infinite q‐factors,^[^ ^20^, ^21^, ^22^, ^23^ ^]^ unlimited lifetimes,^[^ ^24^, ^25^, ^26^ ^]^ and ultranarrow far‐field resonances.^[^ ^27^, ^28^ ^]^ The sustained electromagnetic energy accumulation within their resonant cavities not only amplifies localized field intensities, but also fundamentally enhances chiroptical interactions at molecular‐electromagnetic coupling scales. This paradigm‐shifting mechanism has already been validated across multi‐band systems spanning visible to mid‐infrared regimes, demonstrating transformative capabilities in biosensing,^[^ ^29^, ^30^, ^31^ ^]^ nonlinear optics,^[^ ^32^, ^33^ ^]^ spin‐selective photon generation,^[^ ^34^, ^35^ ^]^ and quantum spin Hall effects.^[^ ^36^, ^37^ ^]^

Chiral small molecules in biological fluids typically occur at micromolar to submicromolar concentrations, necessitating aqueous‐phase detection.^[^ ^1^ ^]^ However, THz devices exhibit limited detection performance in aqueous environments due to strong aqueous absorption,^[^ ^38^ ^]^ inadequate chirality‐field coupling,^[^ ^9^ ^]^ and polarization detection constraints,^[^ ^39^, ^40^ ^]^ challenging the accurate replication of clinical testing conditions. In this context, microfluidics^[^ ^41^, ^42^, ^43^, ^44^ ^]^ offers a promising solution for aqueous THz biosensing, enabling microliter‐scale sample processing, reducing solvent polarity effects, and enhancing detection sensitivity. Integrating metachips with microfluidic systems^[^ ^45^, ^46^ ^]^ minimizes aqueous background absorption through reduced sample volumes, enhancing THz information extraction from analytes,^[^ ^47^ ^]^ thus advancing aqueous‐phase biosensing. However, limited channel multiplicity constrains resolution, and research in recent years has increasingly focused on high‐throughput screening.^[^ ^48^, ^49^, ^50^, ^51^ ^]^ Yet, the critical need for systematic verification through biological sample testing^[^ ^12^, ^52^ ^]^ remains inadequately addressed. This lack of thorough validation experiments poses a significant barrier to establishing methodological reliability and advancing clinical applications.

**Figure 1:**
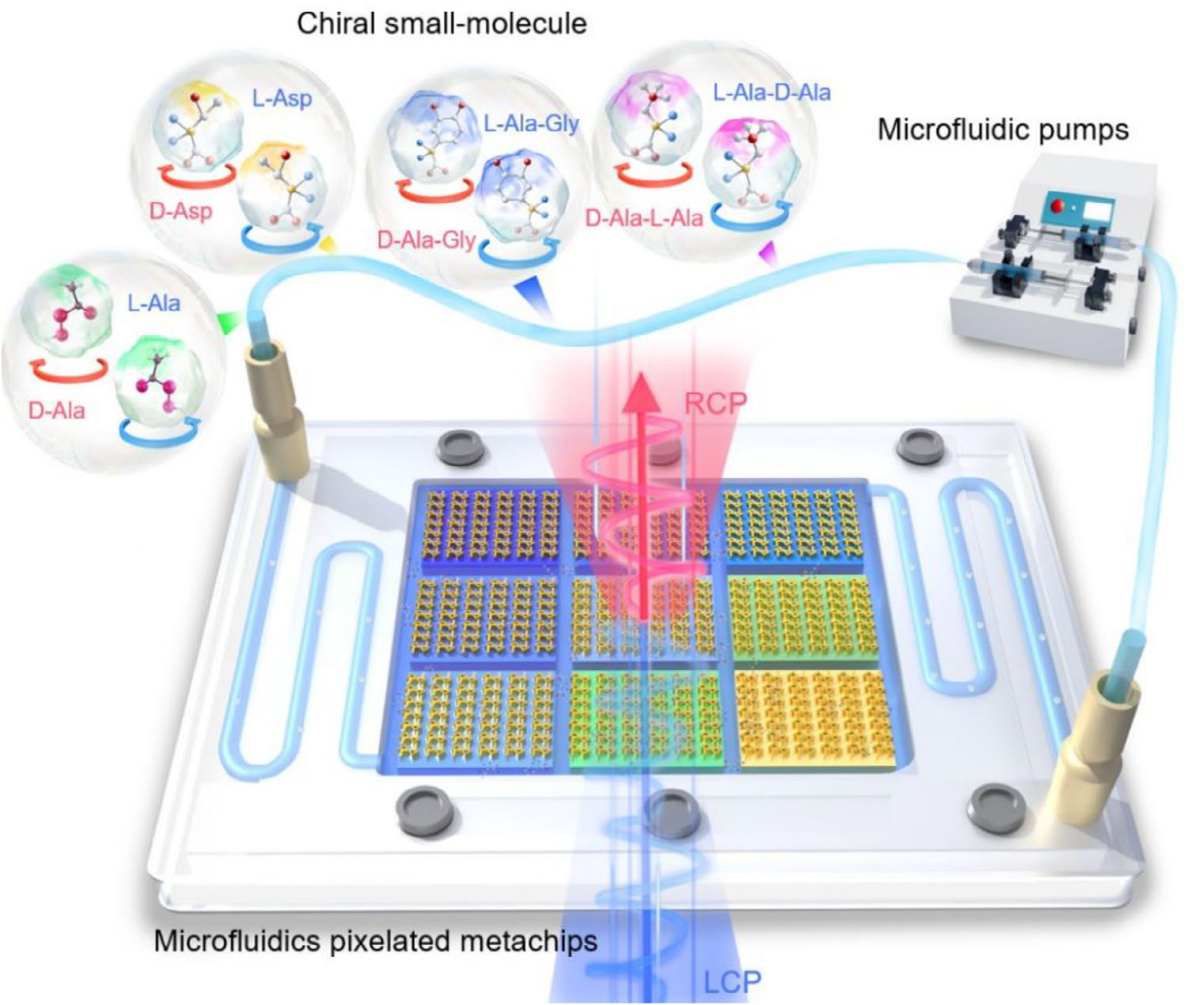
The conceptual representation of the proposed chiral q‐BIC‐microfluidic biosensing platform. The sidewall‐integrated pixelated metachip within the microfluidic cavity enables enantiomer discrimination. Chiral analytes flow through Polytetrafluoroethylene (PTFE) tubes with precision flow control. Under dual‐helical THz wave incidence, symmetry‐broken pixels generate CD spectra via resonant polarization conversion, enabling molecular fingerprinting of aqueous enantiomer solutions.

To address these limitations, we propose an ultra‐compact microfluidic biosensing platform integrating a THz chiral metachip (**Figure** **1**), which enables label‐free, high‐sensitivity, high‐throughput, and rapid identification of chiral enantiomers in aqueous environments. The metachip's 2D pixel array achieves background‐clean CD resonance spectra via q‐BIC excited by breaking in‐plane *C*_4_ symmetry, thus providing distinct molecular fingerprints. Microfluidic integration allows real‐time aqueous monitoring, while dimensionality reduction strategies compress THz spectral signatures into 2D feature vectors, significantly enhancing chiral discrimination accuracy and concentration quantification. Using four pairs of chiral molecules, our platform exhibits a record sensitivity of 200.24 GHz RIU^−1^ and discrimination accuracy of 87.5%, demonstrating substantial potential for early disease diagnosis and drug development.

## 2 Design of BIC‐Based Chiral Metadivic

**Figure 2:**
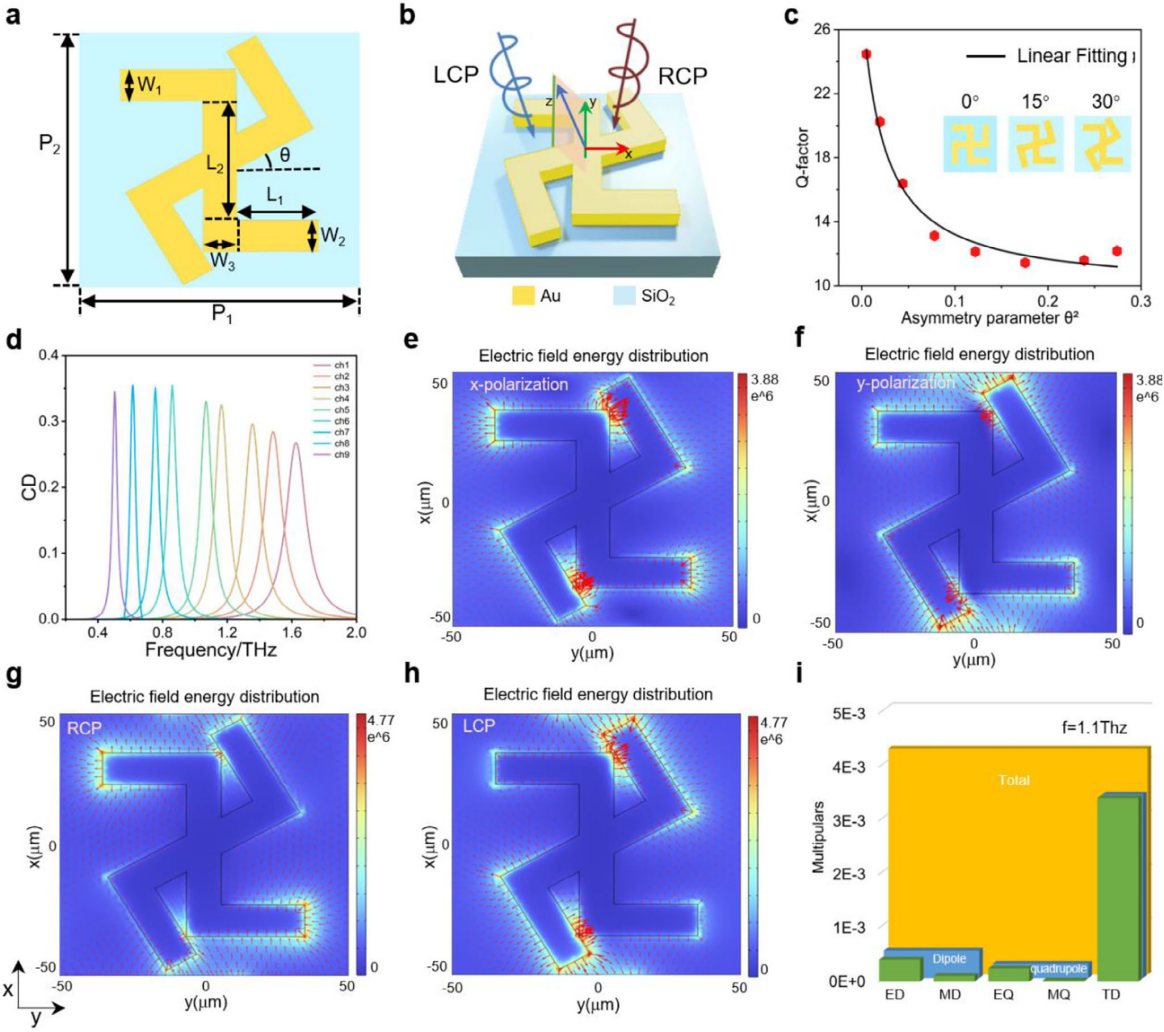
Characterization of q‐BIC meta‐chip and biosensing performance metrics from simulations and measurements. a) Schematic of *C*_4_ rotational symmetry‐breaking for BIC to planar chiral q‐BIC transformation. b) Unit cell of planar chiral q‐BIC composed of two centrally aligned gold Z‐shaped structures on SiO_2_ substrate. c) *Q*‐factor scaling with asymmetry parameter *θ* ^2^. d) Multi‐channel CD transmission spectra for nine sensing units (frequency window: 1.0–1.2 THz). e–h) Simulated Electric field distributions of the blank metachip for linear polarization (LP) and circular polarization (CP) incident waves at 1.1 THz. i) Multipole decomposition at 1.1 THz.

The metachip features a 3 × 3 meta‐pixel array fabricated by a gold lift‐off process, with each unit integrating two rotation‐tunable *Z*‐shaped gold structures (rotation angle *θ*) on 200 µm quartz glass (n = 1.96). This substrate thickness yields 60% volume reduction versus conventional THz sensors (500 µm^[^ ^12^ ^]^). The Critical dimensions of the smallest unit cell: *P*_x/y_ = 100 µm, *L*_1_ = 29 µm, *L*_2_ = 52 µm, *W*_1_ = *W*_2_ = *W*_3_ = 12 µm, gold thickness *H* = 200 nm (**Figure** **2**a). Controlled *θ*‐induced symmetry breaking (0° ≤ *θ* ≤ 30°) disrupts in‐plane *C*_4_ symmetry (Figure 2b), exciting chiral q‐BIC modes with high‐*Q* resonance. Chiral discrimination capability is quantified by *T*_CD__:_

(1) $$T_{\mathrm{CD}}={\left|P_{r}\right|}^{2}+{\left|C_{r}\right|}^{2}-\left({\left|P_{r}\right|}^{2}+{\left|C_{l}\right|}^{2}\right)$$

where subscripts *r*/*l* denote right/left circularly polarized incident light, with *P_i_* and *C_i_* (*i* = *r, l*) representing the co‐polarized and cross‐polarized transmission coefficients, respectively. This corresponds to the Jones matrix in the circular basis:

(2) $$J_{\mathrm{cric}}=\left(\begin{array}{c} P_{r}\;C_{l}\\ C_{r}\;P_{l} \end{array}\right)$$

As the rotation angle θ increases from 0° to 30°, progressive *C*_4_ symmetry breaking occurs (complete at *θ* = 30°), giving rise to enhanced cross‐polarization conversion efficiency, narrowed CD spectral linewidth (Δ*f* = 0.15 THz at peak CD = 0.42). As detailed in our comparative analysis (**Table** **1**; Figure S1, Supporting Information), this combination of strong CD and narrow linewidth surpasses the performance of previously reported planar THz platforms. The resonant properties of the metachip were simulated using a single unit cell model with periodic boundary conditions. The validity of this approach was confirmed by supercell simulations, which showed that inter‐element coupling is negligible (Figure S2, Supporting Information). The chiral q‐BIC's *Q*‐factor follows the inverse quadratic dependence *Q ∝ θ* ^−2^ (Figure 2c), providing a convenient way to tailor the *Q*‐factor of the chiral response. Leveraging electromagnetic scaling invariance, resonant frequency shifts linearly with unit cell size adjustments, facilitating spectral tuning across chiral analytes. Figure 2d maps the simulated resonant frequencies and CD amplitudes for each of the nine sensing channels.

**Table 1:** Survey of reported THz biosensing platforms.

Ref.	Working frequency	CD	Q	RIU	Posteriori experiment	Channels	Years
3D out‐of‐plane chiral plasmonic metasurface^[^ ^10^ ^]^	0.40–1.28µm	0. 45	/	761 nm RIU^−1^	No	1	2022
Flexible thin‐film metal metamaterial^[^ ^11^ ^]^	0.8–1.8THz	/	14.2	243 GHz RIU^−1^	No	1	2020
metal metamaterial^[^ ^12^ ^]^	0.2–1THz	/	6.91	124 GHz RIU^−1^	No	1	2018
Twisted metamaterial^[^ ^53^ ^]^	0.3–0.8THZ	0.15	/	/	No	1	2022
Coupling‐enabled chiral metasurface^[^ ^54^ ^]^	0.66THz	0.16	/	/	No	1	2023
Twisted‐layered chiral metachips^[^ ^55^ ^]^	1.4–1.5 THz	0.6	/	/	No	4	2020
Out‐of‐plane symmetry breaking metachip^[^ ^56^ ^]^	10.8–11.2THz	0.99	10^8^	/	No	1	2024
E‐shaped gold metachip^[^ ^57^ ^]^	0.1–0.7THz	0.525	4.45	39.4 GHz RIU^−1^	No	1	2025
Our work	0.5–2 THz	0.43	28	200 GHz RIU^−1^	Yes	9	2025

Figure 2e–h maps the electric field distributions at 1.1 THz under distinct incident polarizations. *x*‐Polarized excitation concentrates energy at the central junction of the double‐*Z* monomer, while *y*‐Polarized wave**s** localize dipole resonances at the outer corners of the *Z*‐arms. For CP, orthogonal field coupling simultaneously activates both resonance zones under right/left hand circular polarization (RCP/LCP) incidence, generating rotationally opposed induced currents (counterclockwise/clockwise). Chiral samples were modeled as Lorentz oscillators with opposite handedness at 1.1 THz resonance:

(3) $$\varepsilon_{l}\left(\omega \right)=\varepsilon_{l0}+\frac{\Delta \varepsilon_{l}\omega_{0}^{2}}{\omega_{0}^{2}-\omega^{2}-j\omega \delta_{0}}$$

(4) $$\varepsilon_{r}\left(\omega \right)=\varepsilon_{r0}+\frac{\Delta \varepsilon_{r}\omega_{0}^{2}}{\omega_{0}^{2}-\omega^{2}-j\omega \delta_{0}}$$

where *ε*_l0_ and *ε*_r0_ denote the non‐resonant background permittivity, *ω*₀ = 2π × 1.1 THz is the resonant angular frequency, *δ*₀ = 1.0021 × 10^11^ rad s^−1^ the damping rate, and Δ*ε* the oscillator strength. Chirality matching between the metachip and molecules induces enantiomer‐selective resonance: the L‐chiral sample exhibits a stronger response to LCP incidence, while the D‐chiral sample demonstrates a stronger response to RCP incidence (Figure S3, Supporting Information). Discrimination of chiral enantiomers is primarily based on the spatial structural differences of molecules. The metachip magnifies these differences by exciting rotating electric fields with opposite handedness under LCP and RCP incident waves, enabling precise enantiomer identification.

To elucidate chiral metachip resonance modes theoretically, we quantified multipole scattering intensities via electromagnetic simulations (Figure 2i). The results demonstrate that under the chiral q‐BIC resonance regime, the electric dipole (ED) and toroidal dipole (TD) modes exhibit more pronounced resonance peaks compared to other multipoles. Specifically, at *f* = 1.1 THz, the contributions of both ED and TD reach their maximum values, indicating a strong excitation of electromagnetic dipole resonance at this frequency. Further numerical analysis reveals that ED modes primarily govern co‐polarized transmission due to asymmetric charge distribution‐induced dipole moments, while TD modes dominate cross‐polarized transmission through vortex‐like magnetic fields generated by closed‐loop toroidal currents. These multipolar resonances synergistically establish the structure's chiral response mechanism.

**Figure 3:**
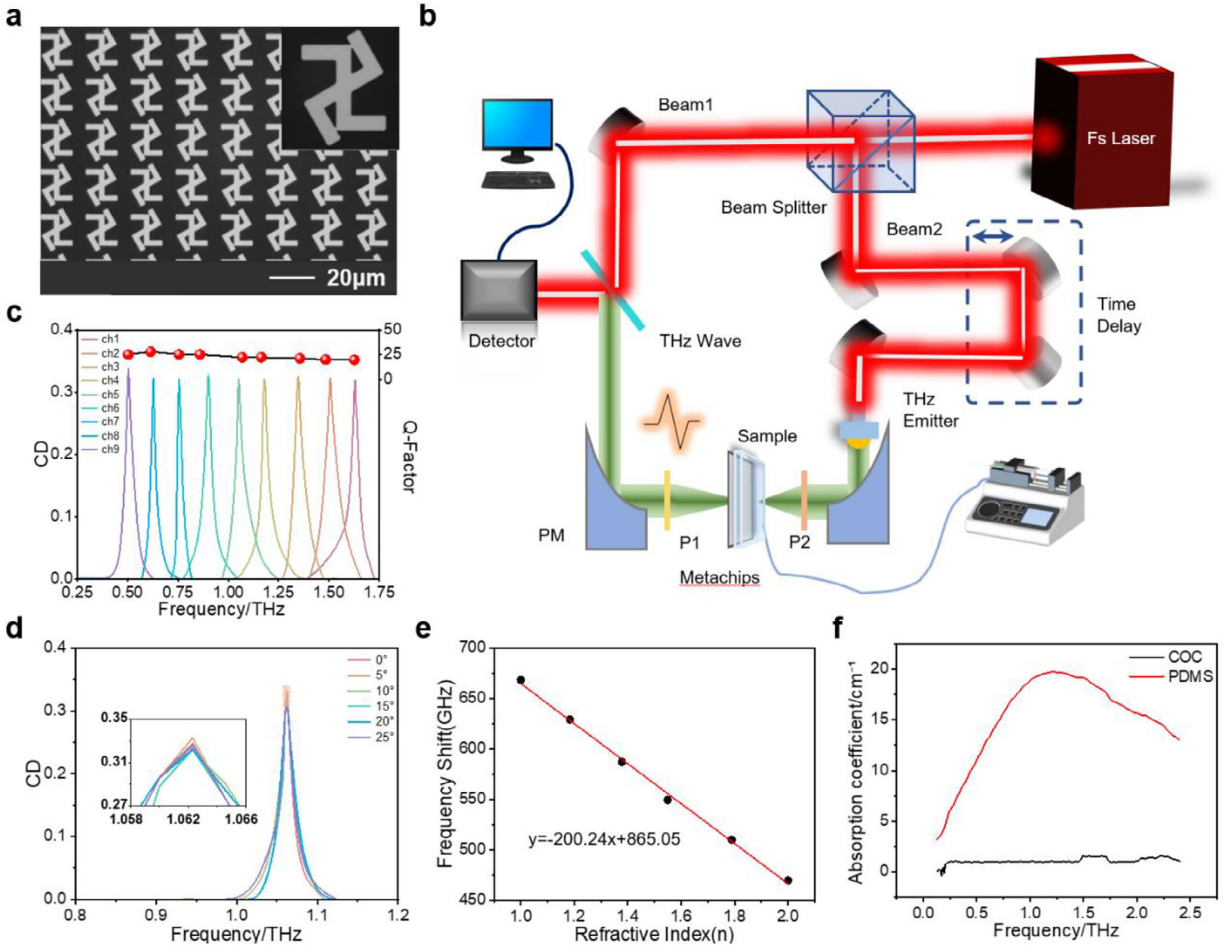
Construction of the experimental system and characterization of performance. a) SEM images of fabricated metapixels and meta‐atoms b) Experimental optical path for THz‐TDS measurements, with integrated microfluidic. c) Measured CD spectra with Q‐factors. d) Angle‐dependent CD spectral evolution on incident angles α = 0–30° e) Refractive index sensitivity calibration curve (200.24 GHz RIU^−1^). f) THz absorption coefficients of COC versus polydimethylsiloxane (PDMS) (0.1–2.4 THz).

To experimentally validate the chiral q‐BIC mechanism, optical microscope was employed to characterize the fabricated metachips (**Figure** **3**a), revealing gold nanostructures conforming to design specifications with uniform morphology. Electromagnetic responses were measured using a transmission‐mode THz‐TDS system. Addressing the system's inherent limitation of LP output/detection, positioned polarizers on both sides of the sample were integrated into the optical path. By varying the incident polarization state and polarizer angles, four LP transmission components are recorded to reconstruct CD, quantifying chiral differential transmission (Figure 3b). For aqueous‐phase sensing, a cyclic olefin copolymer (COC) microfluidic chamber was integrated, with syringe pumps precisely modulating flow rates to maintain steady analyte flow. Signal detection was synchronized via a lock‐in amplifier coupled to a data acquisition module, enabling real‐time monitoring and analysis. The measured CD spectra (Figure 3c) show uniformly shifted resonant peaks within the 0.5–1.75 THz range, achieving a *Q*‐factor of 28, a value compared to previous THz chiral sensors. The specific measured and simulated resonance frequency values of various channels can be found in Table S1 (Supporting Information). While the measured and simulated CD spectra exhibit good agreement, the Q‐factors’ measured values are lower than the simulated ones. This reduction can be attributed to several factors, including the intrinsic metallic loss and the inherent deviation between the idealized simulation and the physically fabricated device. Our model assumes perfect geometries and non‐dispersive material parameters, whereas the fabricated device inevitably possesses imperfections, such as slight variations in critical dimensions and surface roughness, which can introduce additional, unmodeled scattering and absorptive losses. In addition, the system used in our work employs a displacement stage performing rapid scanning to capture spectral data from various channels. This method ensures that for a given setting, the variations in the test environment between the sequential measurements of each channel are negligible. Despite this reduction in Q‐factor, the device maintains excellent performance for multi‐channel analysis. The spacing between resonant peaks across individual pixels exceeds 0.1 THz, surpassing the system resolution limit and guaranteeing unambiguous spectral identification.

To evaluate the angular stability, we illuminated the structure with CP light (LCP/RCP) at various angles of incidence. Both numerical simulations (Figure S4a, Supporting Information) and experimental measurements (Figures 3d; S4b, Supporting Information) confirm that the CD value remains consistently above 0.3 for incident angles ranging from 0° to 30°. This perfect angular robustness is a hallmark of the structure's intrinsic chirality, confirming that the chiroptical response is a fundamental property of the geometry itself, rather than an extrinsic effect dependent on specific, symmetry breaking illumination angles. This stability arises from the high *Q*‐factor of the q‐BIC mode, which suppresses angle‐dependent distortions. The sensing mechanism is based on resolving local refractive index changes from a molecular adlayer, which are governed by the analyte's intrinsic properties like size and chiroptical activity. Simulations establish a linear redshift of 200.24 GHz RIU^−1^ while maintaining CD > 0.3(Figure 3e) and stabilizing the *Q*‐factor at 22 (Figures S5, Supporting Information). The Q‐factor provides the spectral resolution to detect molecular binding, while the CD provides the differential signal to distinguish enantiomers. This combination confirms the device's ability to translate subtle, enantiomer‐specific properties into a clear and measurable spectral signature.

Recognizing the absorption effect of aqueous molecules in the THz band, the inherent height of the metachip (*h* = 200 µm), and interference from cavity bubbles, we developed a reusable microfluidic q‐BIC chiral sensing chip (Figure S6, Supporting Information). This chip enables high‐throughput aqueous sample detection and supports rapid metachip replacement via modular side interfaces. By employing COC substrate for its superior THz transparency, we measured absorption coefficients (0.01–1 cm^−1^, 0.1–2.4 THz) 1–2 orders lower than PDMS (peak absorption ≈20 cm^−1^; Figure 3f). We further constructed a THz time‐domain spectroscopy platform that integrated the microfluidic system. Critically, contact angle measurements (Figure S7a, Supporting Information) validate chiral molecule adsorption onto the gold metachip: differential contact angles indicate that 0.05 mg dL^−1^ solution concentrations facilitate binding to Au surfaces.

## 3 Experimental Validation Using Chiral Molecule Samples

We further analyzed the fingerprint spectra of chiral small molecules—L/D‐Alanine, L/D‐Aspartic Acid, L‐Ala‐D‐Ala/D‐Ala‐L‐Ala, and L/D‐Ala‐Gly (solution details in the Experimental Section) in PBS at concentrations of *C*₁ = 0.05, *C*_2_ = 0.15, and *C*_3_ = 0.3 mg dL^−1^. Taking Channel 1 as an example, significant resonant frequency shifts were induced by D‐Alanine and both enantiomers of Ala‐Gly, while minimal shifts were observed for other molecules. Consistent with the refractive index‐frequency relationship in Figure 2e, which indicates higher effective refractive indices for D‐Alanine and L/D‐Ala‐Gly at Channel 1. Before examining chiral signatures, we highlight the metamaterial chip's exceptional concentration sensitivity (Δ*f*/Δ*C*) documented in Figure S7b (Supporting Information): L‐Aspartic (0.8312), D‐Aspartic (0.80728), L‐Alanine (0.38508), D‐Alanine (1.8192), L‐Ala‐Gly (1.4996), D‐Ala‐Gly (2.1024), L‐Ala‐D‐Ala (1.0444), D‐Ala‐L‐Ala (1.26944) THz·mg^−1^·dL. Specifically, D‐Ala‐Gly showed a peak sensitivity of 2.1024 THz·mg^−1^·dL, surpassing the prior maximum of 1.44 THz·mg^−1^·dL for label‐free THz biosensors.^[^ ^58^ ^]^

**Figure 4:**
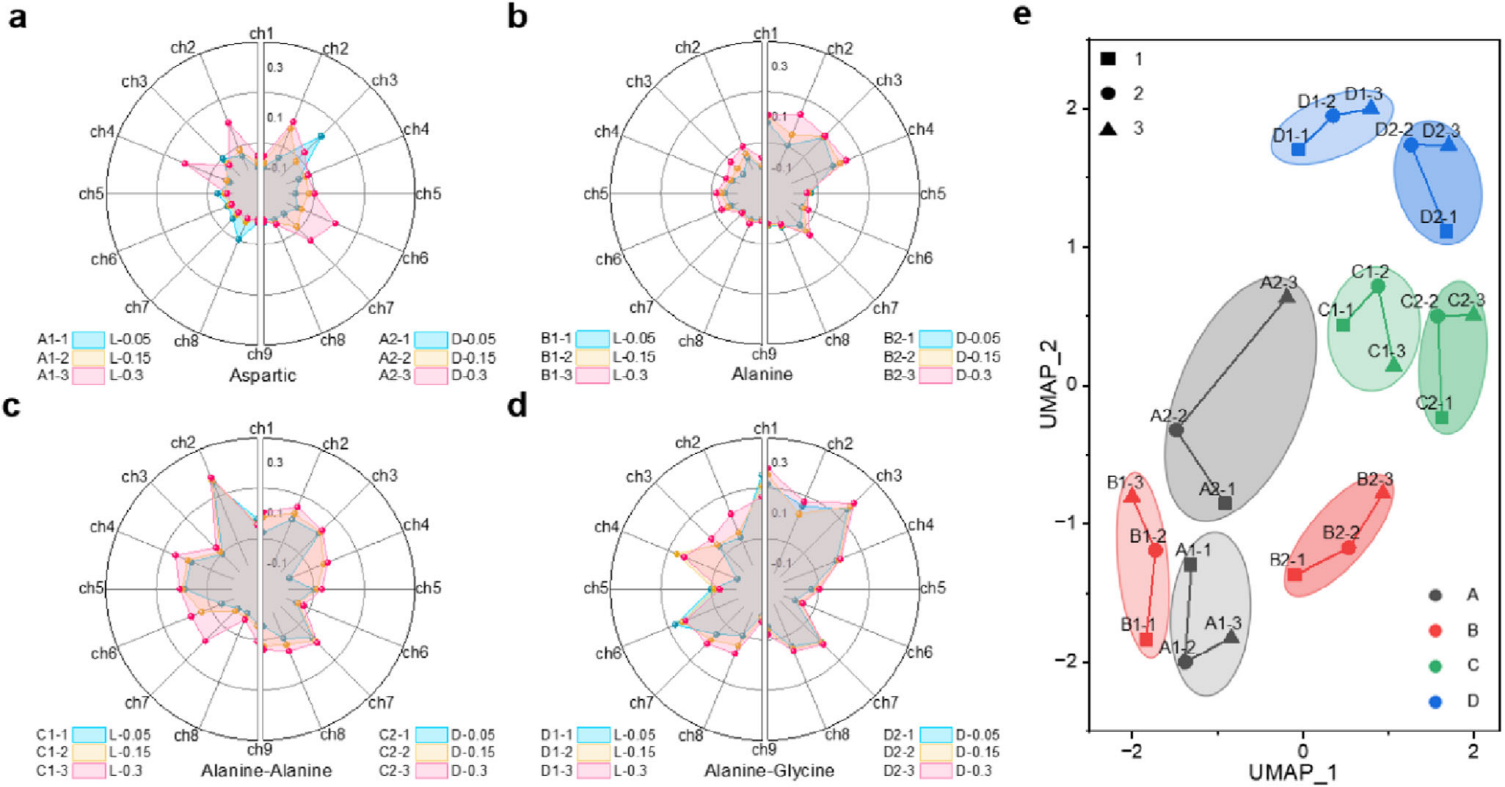
High‐dimensional chiroptical signatures and UMAP‐based feature extraction in chiral molecular. a) Radar charts displaying the CD spectra of L/D‐Aspartic acid at varying concentrations. b) Radar charts displaying the CD spectra of L/D‐Alanine at varying concentrations. c) Radar charts displaying the CD spectra of L‐Ala‐D‐Ala / D‐Ala‐L‐Ala dipeptide at varying concentrations. d) Radar charts displaying the CD spectra of the L/D‐Ala‐Gly dipeptide at varying concentrations. e) 2D spatial distribution of chiral molecular data points, visualized by UMAP.

As depicted in **Figure** **4**a–d, the resonant frequency shifts (Δ*f*) from nine channels (ch1–ch9) form discriminative feature vectors that capture concentration‐dependent responses to chiral molecules across *C*₁ = 0.05, *C*_2_ = 0.15, and *C*_3_ = 0.3 mg dL^−1^. The complete THz CD spectra underlying these radar plots are archived in Figures S8–S10 (Supporting Information). This multi‐channel heterogeneity in responses validates the hyperspectral chiral sensing capability, extending label‐free detection sensitivities. From a physical perspective, such optical responses arise when aqueous media are modeled as effective complex refractive index systems (*n* = *n*' + *jn*“”), where the constituents (*n*', *n*“”) are modulated by molecular conformations, electrostatic interactions, concentration gradients, solvent dielectric properties, and ambient temperature.

To reduce the dimensionality of raw spectral features and enable effective visualization, we employed UMAP to project high‐dimensional THz spectral data into a 2D space, as shown in Figure 4 (Details can be found in the Experimental Section). This algorithm preserves intrinsic topological invariants by constructing fuzzy simplicial complexes from local metric approximations, followed by global cross‐entropy minimization on Riemannian manifolds. The resultant embedding coordinates (*x*, *y*) are dimensionless, reflecting relative positional relationships—proximity indicates intrinsic data similarity. The 2D projection exhibits resolved cluster segregation, with distinct domains occupied by each stereoisomer (L/D) concentration level. This facilitates reliable classification and concentration‐dependent analysis, significantly enhancing throughput efficiency via feature‐space compression (9D→2D) while maintaining sub‐mg/dL sensitivity.

**Figure 5:**
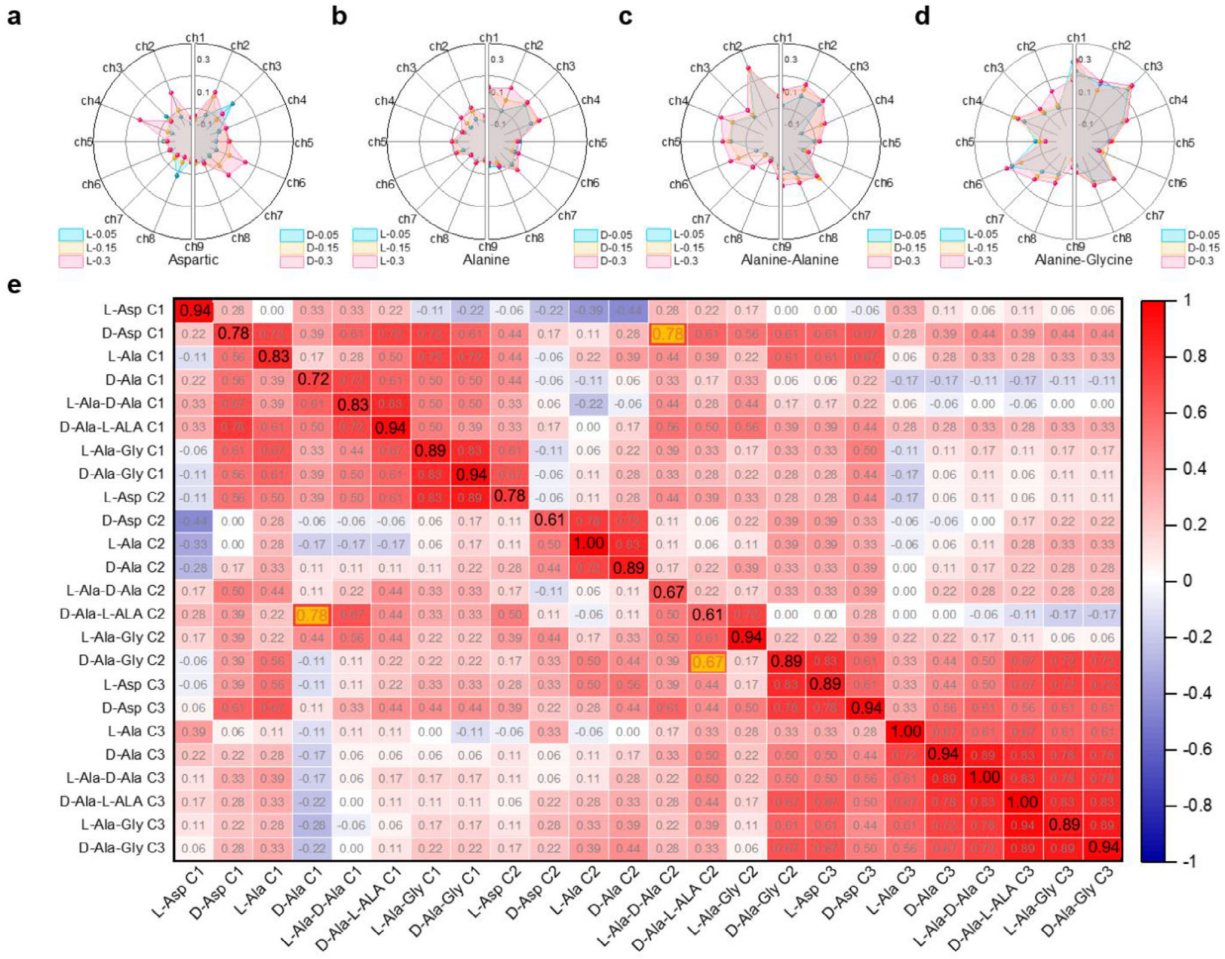
Validation of THz CD spectral signatures and detection efficacy using extended chiral molecular test samples. a) Concentration‐dependent CD radar plots for L/D‐Asp Acid. b) Corresponding‐dependent CD radar plots for L/D‐Ala Acid. c) Concentration‐dependent CD radar plots for L‐Ala‐D‐Ala / D‐Ala‐L‐Ala dipeptide. d) Concentration‐dependent CD radar plots for L/D‐Ala‐Gly dipeptide. e) Quantitative performance assessment via confusion matrix based on Kendall rank correlation coefficients. Color intensity scales with statistical correlation strength, where the principal diagonal highlights correct predictions while off‐diagonal elements indicate classification errors.

Furthermore, to benchmark the robustness of the dimensionality‐reduced feature space, we evaluated a new test set comprising all target chiral small molecules across their concentration gradients. The UMAP‐derived molecular fingerprints for these samples are presented in **Figure** **5**a–d, while raw THz CD spectra reside in Figures S11–S13 (Supporting Information). Kendall rank correlation‐based confusion matrix analysis (Figure 5e) validates 87.5% species identification accuracy (details see Experimental Section). A closer examination reveals that the misclassifications are primarily due to the high structural similarity between certain analytes, such as the confusion observed between D‐Ala‐L‐Ala and D‐Ala. The classification robustness could be further improved by increasing the number of sensing channels. This would expand the feature dimensionality and enhance the correlation contrast between these closely related molecules, thereby improving the method's resolution and reliability.

## 4 Conclusion

In summary, we developed a label‐free THz microfluidic metachip for ultrasensitive, high‐throughput detection and quantification of chiral small molecules in aqueous media. This platform overcomes THz wave attenuation by simultaneously mitigating substrate interference and aqueous absorption. The implementation of dimensionality‐reduced 2D spatial projections of spectral features enables unambiguous molecular identification, eliminating the need for spectral assignment expertise and thereby expediting solution‐phase chiral analysis. Systematic validation with independent test sets demonstrated 87.5% classification accuracy and operational robustness. Furthermore, inter‐batch reproducibility was evidenced by replicated experiments with chiral analyte‐laden PBS solutions, which exhibited consistent spatial distribution under standardized protocols. Future efforts will focus on 3D hyperspectral mapping to enable large‐scale screening of complex biological matrices such as bodily fluids and tissues. While the extension of this methodology to other spectral bands (e.g., infrared and visible) is theoretically possible, its application to specific challenges, such as analyzing chiral molecules, remains complex. The chirality of these molecules often does not produce distinct spectral fingerprints in conventional absorption spectra within these ranges. This inherent lack of specificity complicates the direct identification of chiral molecules and the differentiation of their enantiomers. This cost‐effective methodology, which requires no advanced technical training, establishes a practical platform for chiral biomarker‐based point‐of‐care diagnostics, offering broad utility in disease monitoring and therapeutic assessment.

## 5 Experimental Section

### Main Steps of Sample Fabrication

The metachip fabrication sequence commenced with spin‐coating a 200 µm‐thick layer of positive photoresist (RZJ‐304) onto a quartz glass substrate. UV exposure through a photomask selectively removed irradiated regions of the photoresist, defining the microstructural pattern. Subsequently, a 200 nm‐thick gold layer was deposited on the patterned substrate using a magnetron sputtering system (DSC‐IBSD). Deposition proceeded under an argon atmosphere at 1 Pa pressure, with sputtering powers set to 100 W for Ti and 200 W for Au. The process sequence comprised depositing Ti for 1 min, followed by concurrent sputtering of Ti and Au for another minute, and ultimately sputtering Au for 8 min, yielding final metal layers of Ti (10 nm)/Au (200 nm). Residual metal was removed via lift‐off processing to complete the metachip fabrication.

### Preparation of Chiral Small Molecule Enantiomers in PBS Solution

Commercially sourced HPLC‐grade chiral enantiomers (purity ≥99%, Sigma–Aldrich/TCI) were employed without further purification. The preparation of enantiomeric solutions in phosphate‐buffered saline (PBS) followed a chemically inert protocol: Sterile PBS (0.01 m, 137 mm NaCl, 2.7 mm KCl, pH 7.4) was used as a dissolution medium. High‐purity D/L enantiomers (≥99% ee) were dissolved in sterile PBS at 0.3 mg mL^−1^ to form a stock solution via agitated mixing until optical clarity was achieved, which was then serially diluted (1:1 and 1:5 v/v) to yield 0.15 and 0.05 mg mL^−1^ working solutions. All operations were conducted in amber glass vials under light‐protected isothermal conditions (25.0 ± 0.5 °C) to maintain the stability of the chiral center configuration. The ionic strength (154 mm) of the PBS solution maintains physiological conformational stability of chiral molecules, enabling biochemically relevant interactions during metasurface exposure.

To validate molecular adsorption, the metasurface‐functionalized chip was incubated in test solutions at controlled humidity (45 ± 3% RH) for 1 h, followed by goniometer‐based contact angle measurements. As depicted in Figure S6a (Supporting Information), the contact angles (D‐alanine: 68.3° ± 2.1°; L‐alanine: 65.7° ± 2.5°) demonstrate enantiomer‐specific interfacial interactions at the lowest tested concentration (0.05 mg mL^−1^). These observations corroborate two critical properties: effective binding of chiral molecules to the Au substrate, alongside retention of molecular stereochemical integrity during sample preparation.

### Experimental Settings

The THz measurement system features a 50 mm × 50 mm scanning range with a minimum step resolution of 80 µm (*x*/*y*‐axes), enabling synchronized signal acquisition across metapixels at reduced time cost. Integrated TERAVIL DET‐8 photoconductive antennas (LT‐GaAs type) facilitate real‐time deconvolution of THz signals in chiral small‐molecule PBS samples, generating visualized spectral fingerprints. The system works by measuring spectral shifts from a pre‐calibrated baseline as the analyte flows through the chip's microchannels, enabling multi‐dimensional CD profiling. While validating methodological feasibility, its throughput was limited by the single‐detector configuration of the THz measurement system. However, the array‐based design of the chip inherently supports parallel processing. By integrating the THz system with a detector array and a microfluidic flow (10 µL min^−1^), high‐throughput detection can be achieved within the chiral‐molecule PBS solution system (0.005–0.03 mg mL^−1^).

### The Descending Dimension Method

UMAP was employed for dimensionality reduction, grounded in the manifold hypothesis that high‐dimensional spectral data inherently reside on lower‐dimensional topological manifolds. The algorithm first constructs a fuzzy simplicial complex via k‐nearest neighbor graphs (k = 12), where cosine similarity quantifies local point adjacency and repulsive forces preserve global connectivity. A stochastic gradient descent optimization then embeds this topological structure into a 2D latent space (n_components = 2), with the min_dist parameter (set to 0.35) minimizes local point collapse without distorting inter‐cluster relationships. Hyperparameters were configured as: metric = “cosine” to accommodate amplitude‐invariant spectral similarity, and random_state = 42 to ensure embedding reproducibility. This implementation enables structure‐preserving visualization of chiral molecular spectral clusters while retaining discriminative topological features.

### The Definition of Accuracy

To quantitatively assess the classification performance, a confusion matrix was deployed to analyze the correspondence between predicted labels and ground truth. The predicted label for each sample was determined by the highest predicted probability across all classes. As shown in Figure 5e, 21/24 test samples were correctly classified, with the estimated probability maxima aligning exactly with the diagonal elements of the matrix (highlighted in red), indicating perfect agreement between predictions and true labels. Under this experimental setup, a detection accuracy of 87.5% was obtained, calculated as the ratio of correctly classified samples to the total number of tests. This result suggests the model's robust discriminative capacity for the given task.

## Conflict of Interest

The authors declare no conflict of interest.

## Supporting information



Supporting Information

## Data Availability

The data that support the findings of this study are available from the corresponding author upon reasonable request.
