# Supplementary material for: Multispectral Chiral Quasi‐Bound States in the Continuum Enabled Microfluidics for High‐Throughput Molecular Screening and Quantification
Source: Adv Sci (Weinh). 2025 Sep 28;12(47):e15443. doi: 10.1002/advs.202515443 (PMC12713036; doi:10.1002/advs.202515443)
Supplement: Supplementary file 1 — Supporting Information [file ADVS-12-e15443-s001.docx]

Supporting Information

**Multispectral Chiral Quasi-Bound States in the Continuum enabled microfluidics for high-throughput molecular screening and quantification**

*Xinyue Liang, Zihan Zhao, Xiaocong Tang, Haiyue Yang, Lanju Liang, Meng Zhao, Cong Wang*, Lei Wang*, and Xumin Ding**

**Table of Contents**

Figure S1. Simulated spectra with different angle θ……………………………………………..…2

Figure S2. Simulated results in the 1x3 supercell.…………………………………………….……3

Figure S3. Simulated electric field distributions at 1.1 THz…………………………………..……3

Figure S4. CD spectral with incident angle change.………………………………………...………4

Figure S5. Simulated results of the designed metachip……………………………………..………4

Figure S6. Figure of the measurement system……………………………………………………..5

Figure S7. Molecular selection and experimental validation……………………………………..…6

Figure S8. Transmission CD spectra under 0.05 mg/dL………………………………………….…7

Figure S9. Transmission CD spectra under 0.15 mg/dL……………………………………….……8

Figure S10. Transmission CD spectra under 0.30 mg/dL……………….………………….………9

Figure S11. Transmission CD spectra under 0.05 mg/dL (additional group).………………………11

Figure S12. Transmission CD spectra under 0.15 mg/dL (additional group) ...……………………12

Figure S13. Transmission CD spectra under 0.30 mg/dL (additional group) …...…………………13

1. Calculated results

Figure S1 presents the numerically simulated transmission Jones matrix spectra of the metasurface across rotation angles *θ* (0° to 30°), characterizing its transmitted circular dichroism. Simulations were conducted with commercial electromagnetic software using unit cell periodic boundary conditions in the x and y directions and open (radiation) boundary conditions in the z-direction. The simulations employed a circularly polarized excitation source across a frequency range of 0.5 to 2 THz. The gold layer pattern was described by a conductive lossy metal model (DC conductivity: 4.561×10⁷ S/m) from the material library.


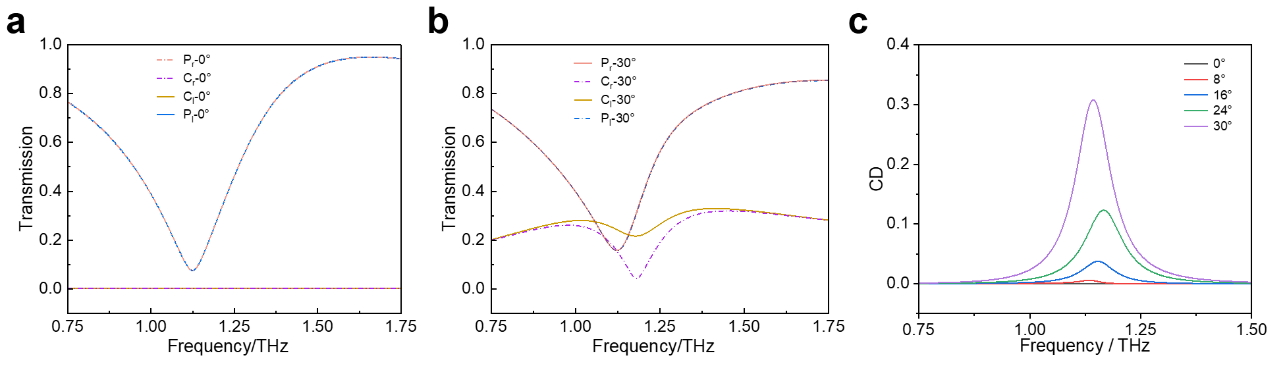


**Figure S1**: Simulated spectra with different angle *θ*. **a b** Simulated transmission Jones matrix spectra of *P*_r_, *C*_r_, *P*_l_, and *C*_l_ spectrum of the meta-surface at *θ* = 0° and *θ* = 30°. **c** The evolution of CD spectra by continuous varying angle *θ*.

To verify that the near-field coupling between adjacent unit cells along the y-axis is negligible, we performed a validation study using a 1x3 supercell model and compared its response to the single unit cell model used in the main text. As shown in Fig. Sn a, the transmission CD spectra are the same as the single unit cell result in Sn. Furthermore, the normalized electric field distribution of the central unit cell in the supercell (Fig. S2 b) is indistinguishable from that of the single unit cell. This confirms that the chosen periodicity is sufficient to prevent inter-element coupling, validating that the use of the single unit cell model is no problem.


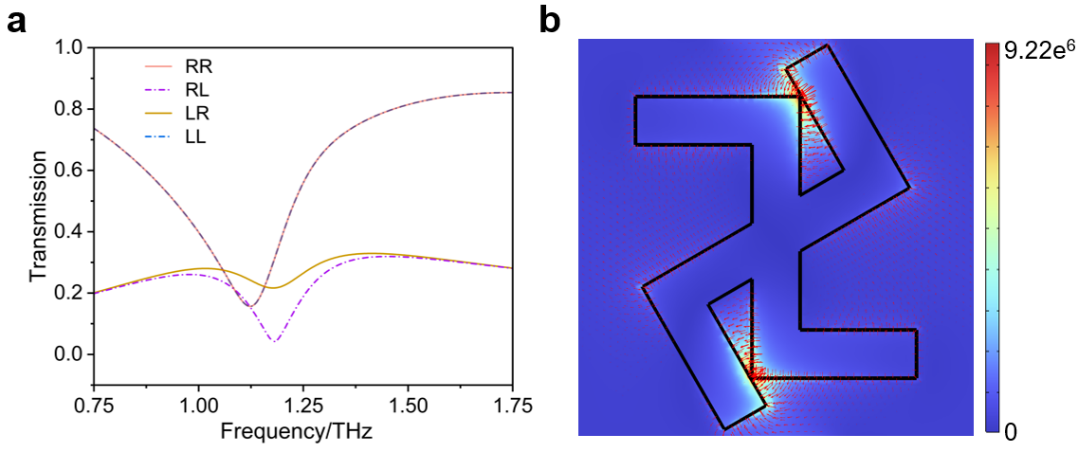


**Figure S2:** Simulated results in the 1x3 supercell. **a** The transmission spectrum from the 1x3 supercell simulation. **b** The E-field plot of the central cell in the 1x3 supercell.

Figure S3 displays the simulated 1.1 THz electric field distributions for the metasurface interacting with L- and D-chiral samples. For a given enantiomer (e.g., D-chiral samples), significant field distribution differences between RCP and LCP illumination indicate the system’s chiral response. Additionally, under identical circular polarization, localized fields show mirror-symmetric distributions for L- and D-enantiomers due to chiral enhancement effects, confirming their opposite chirality.


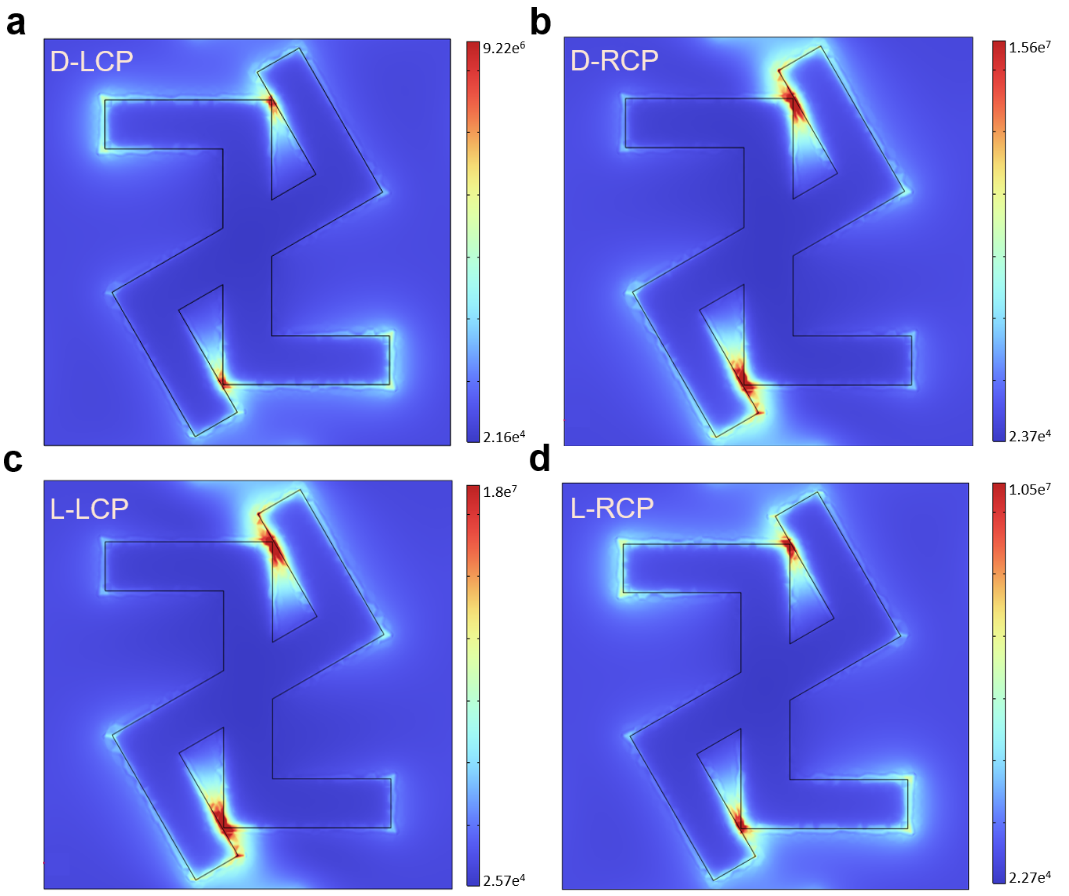


**Figure S3**: Simulated electric field distributions of the metasurface incorporating chiral samples at 1.1 THz. **a** D-chiral sample under LCP incidence. **b** D-chiral sample under RCP incidence. **c**L-chiral sample under LCP incidence. **d** L-chiral sample under RCP incidence.

2. Experimental system and measured results

Table S1 lists the measured and simulated resonance frequencies and corresponding S-parameters. The measured data are in good agreement with the simulated results. Although minor frequency shifts occur due to fabrication tolerances (as shown in Figs. 2d and 3c), they do not interfere with the nearby channels.

**Table 1.** Specific values of simulated and measured resonant frequencies.

| Channels | 1 | 2 | 3 | 4 | 5 | 6 | 7 | 8 | 9 |
| --- | --- | --- | --- | --- | --- | --- | --- | --- | --- |
| *S parameters* | 0.7 | 0.77 | 0.85 | 1 | 1.09 | 1.39 | 1.57 | 1.98 | 2.5 |
| Simulated Results | 1.63 | 1.48 | 1.36 | 1.16 | 1.07 | 0.86 | 0.76 | 0.62 | 0.50 |
| Measured Results | 1.63 | 1.50 | 1.35 | 1.18 | 1.09 | 0.85 | 0.76 | 0.63 | 0.50 |

Figure S4a shows the simulated circular dichroism spectra of the metapixel at incident angles *α* from 0° to 30°. Experimental measurements (Figure S4b setup, results in Figure 3d) agree well with simulations, validating robustness against incident angle variations. The sensor functions by analyte injection: refractive index changes modify the dielectric environment, inducing shifts in resonance characteristics (frequency, absorbance, peak position, etc.).


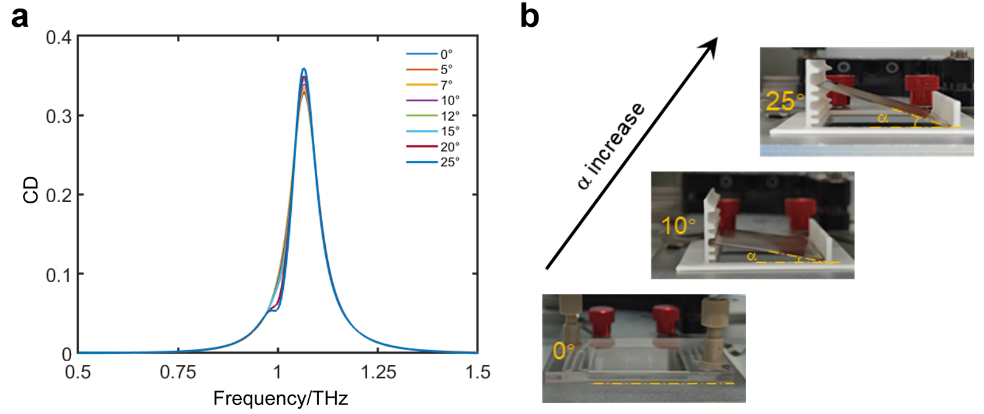


**Figure S4**: **a** Incident angle (*α*=0° - 30°) dependent simulated CD spectral change. **b** Incident angle (*α*=0° - 30°) test platform.

Given that biomolecules typically exhibit refractive indices between 1.0 and 2.0, Figure S4a illustrates the simulated changes in the circular dichroism characteristics of the metapixel upon exposure to such analytes. Sensor performance is evaluated by three metrics: *Q*-factor, sensitivity (S), and figure of merit (FOM). The *Q*-factor, reflecting resonance quality, correlates with structural dielectric losses; higher values yield sharper resonance peaks, thus influencing sensitivity and resolution. The FOM, quantifying overall performance (higher values indicating superiority), is calculated as:

$$Q=\frac{f}{FWHM}$$

$$FOM=\frac{S}{FWHM}$$

where *f* is the resonance frequency and FWHM (full width at half maximum) the spectral linewidth. Simulation results appear in Figures S5b and S5c.


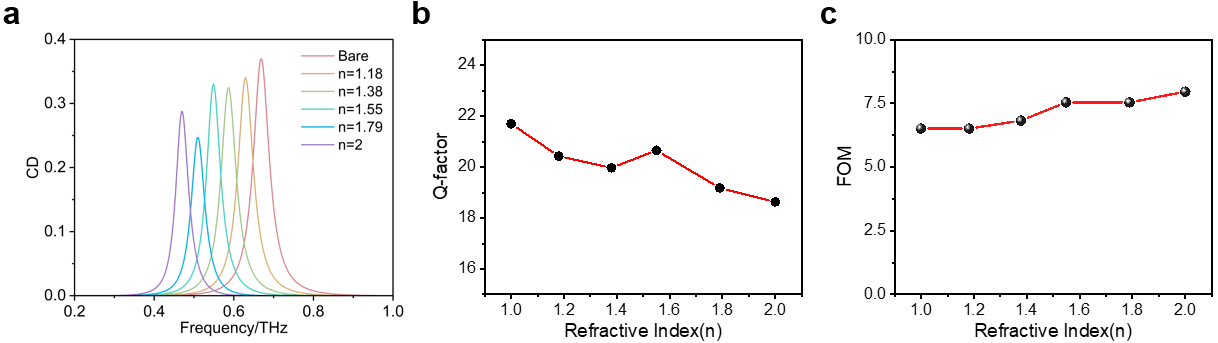


**Figure S5**: **a** Simulated CD spectra for different refractive indices. **b** Effect of analytes refractive indices on *Q*-factor. **c** Effect of analytes refractive indices on FOM.

To explore clinical utility, we integrated an open-aperture terahertz measurement system with a computational backend (equipped with analysis software) into a microfluidic chip, enabling rapid analysis of fluid samples (Figure S6a). A custom microfluidic chip interfaced with the metachip, connected via polytetrafluoroethylene (PTFE) tubing to a microfluidic pump, enabled precise delivery of solution samples for metachip interaction and raw spectral data acquisition (Figures S6b, c). To validate performance, we tested 24 samples in a 2D spatial map: eight chiral small-molecule solutions at three concentration levels. Each concentration solution required ~30 min for chip flow/interaction, with ~1 min per sample per channel for spectral acquisition. Total dataset acquisition (including system debugging and software calibration) took ~18 hours.


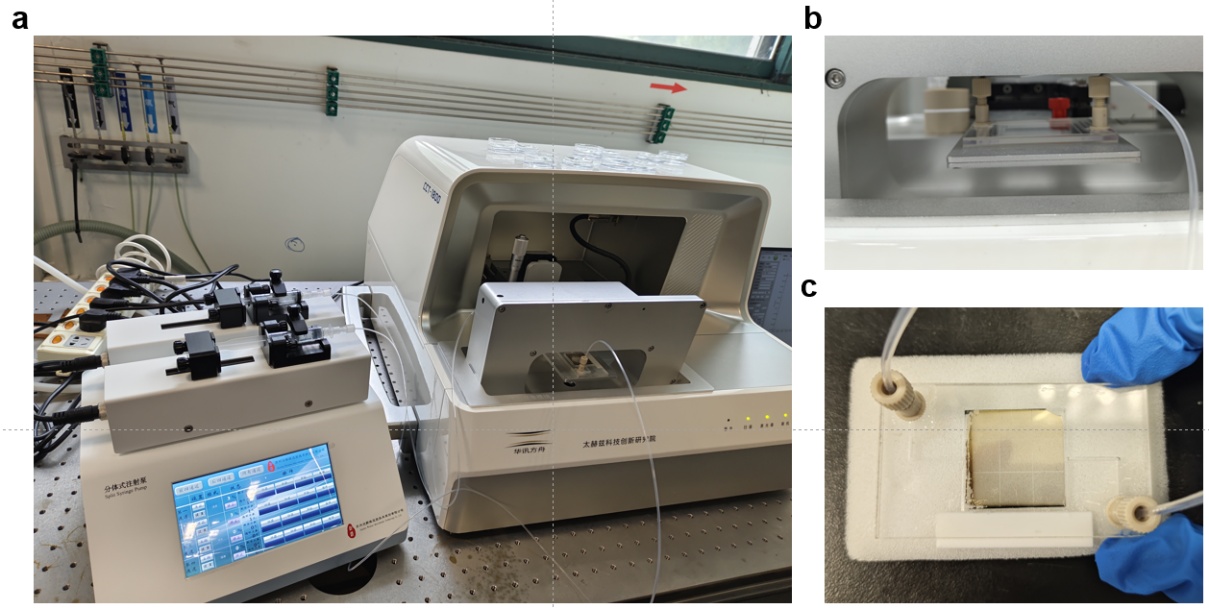


**Figure S6**: Figure of the measurement system. **a** Photograph of the THz measurement system integrated with the microfluidic setup. **b** The metachip integrated with the microfluidic chip, positioned on the scanning translation stage. **c** Photograph of the fabricated microfluidic chip.

To validate chiral molecule adsorption on the metallic metachip, contact angle measurements were performed (Figure S7a). Chiral molecules were dissolved in PBS buffer to prepare solutions of defined concentrations, followed by metachip immersion for 1 hour and subsequent contact angle analysis. Owing to the inherent variability of contact angle measurements, enantiomer discrimination was not feasible; these results thus serve as supplementary evidence only.

To evaluate concentration sensitivity, we defined sensitivity as *U*=Δ*F*/(*C*_3_−*C*_1_), where Δ*F* is the frequency shift, and *C*_1_ and *C*_3_ denote the lowest and highest concentrations, respectively. Sensitivity results for the metachip with different chiral molecule solutions are presented in Figure S7b.

**
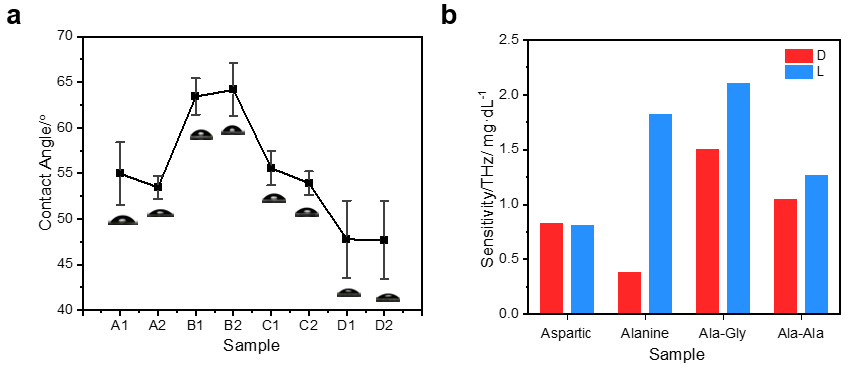
**

**Figure S7**: **a** Contact angle test results. **b** Sensing performance index of the designed metachip.

3. Experiments on a group of chiral small molecule solutions.

Figures S8 through S10 illustrate the changes in the circular dichroism spectra resulting from the integration of the chiral small molecules at three distinct concentrations with the designed metachip. To differentiate the circular dichroism spectra corresponding to the various resonant channels, different colors have been employed. Notably, vertical dashed lines indicate the resonant frequencies observed following the introduction of the chiral small molecule solutions.

**
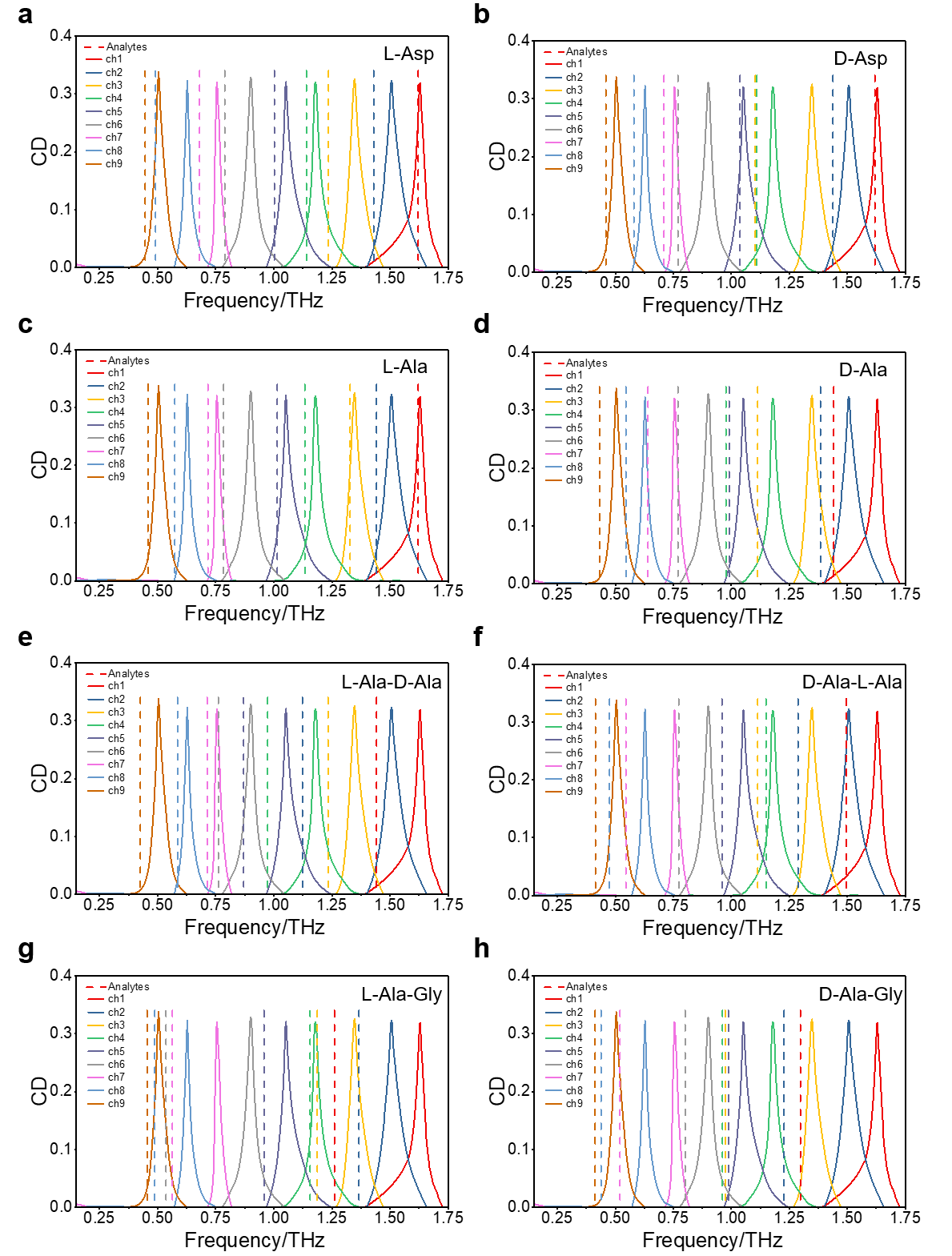
**

**Figure S8**: **Changes in multichannel terahertz CD after a mixture of different chiral molecular solutions at a concentration of 0.05 mg/dL (C_1_). a** L-Aspartic. **b** D-Aspartic. **c** L-Alanine. **d** D-Alanine. **e** L- Alanine - D - Alanine. **f** D- Alanine - L - Alanine. **g** L-Ala-Gly .**h** D-Ala-Gly.

**
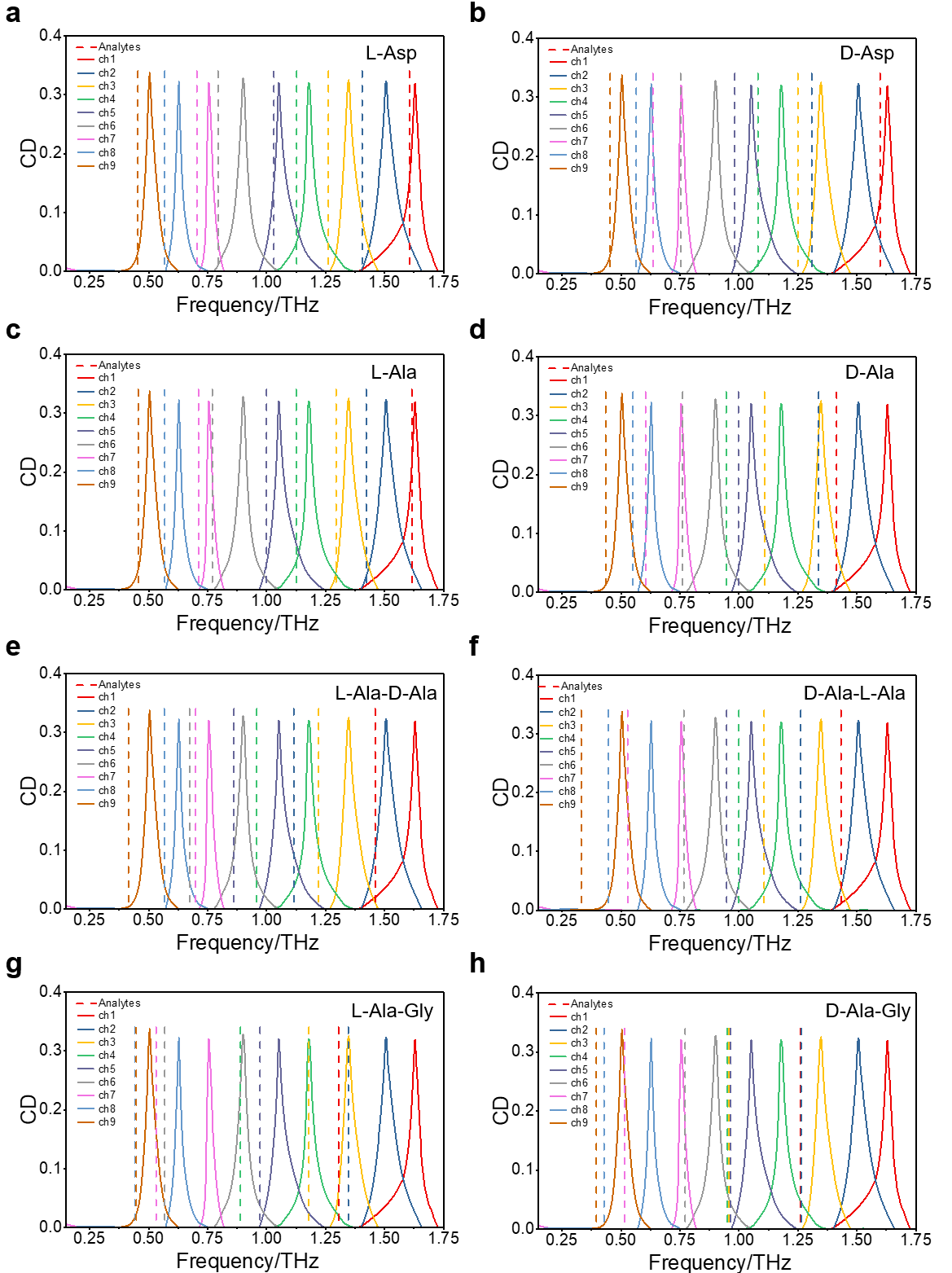
**

**Figure S9**: **Changes in multichannel terahertz CD after a mixture of different chiral molecular solutions at a concentration of 0.15 mg/dL (C_2_). a** L-Aspartic. **b** D-Aspartic. **c** L-Alanine. **d** D-Alanine. **e** L- Alanine - D - Alanine. **f** D- Alanine - L - Alanine. **g** L-Ala-Gly .**h** D-Ala-Gly.

**
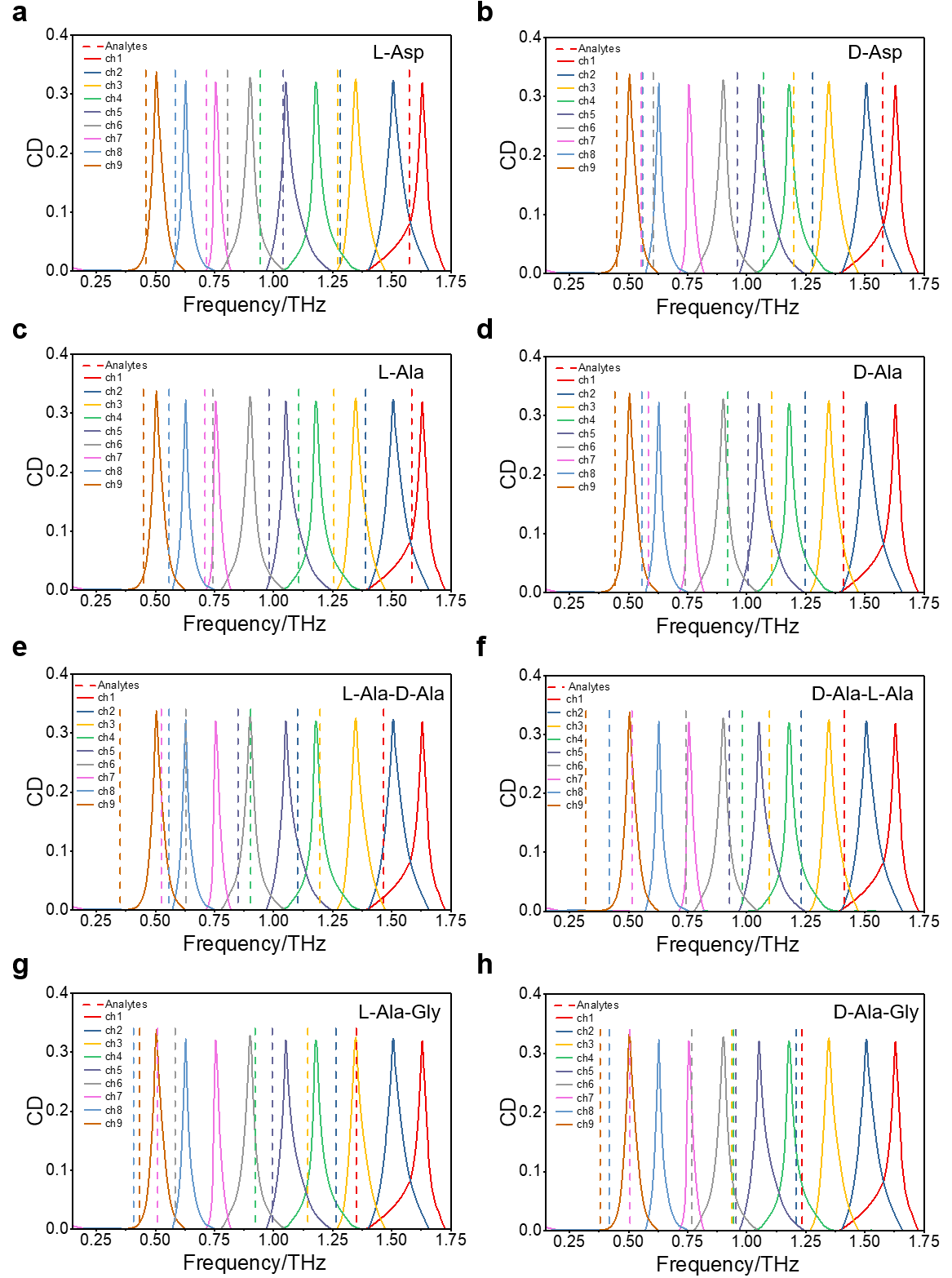
**

**Figure S10**: **Changes in multichannel terahertz CD after a mixture of different chiral molecular solutions at a concentration of 0.3 mg/dL (C_3_). a** L-Aspartic. **b** D-Aspartic. **c** L-Alanine. **d** D-Alanine. **e** L- Alanine - D - Alanine. **f** D- Alanine - L - Alanine. **g** L-Ala-Gly .**h** D-Ala-Gly.

4. Experiments with an additional group of chiral small molecule solutions.

To ensure output reliability, we controlled for extraneous factors unrelated to the detection process. To evaluate the analytical pipeline’s accuracy, we used an independent set of biological samples prepared via the identical protocol. This set includes a diverse range of chiral small molecules in 2D spatial maps (Figure 4e). Figures S11–S13 show circular dichroism (CD) spectral changes observed after incubating these samples with the metachip, from which characteristic spectral fingerprints were extracted via baseline-normalized differential analysis (with blank-chip measurements as reference), depicted in the radar charts in Figure 5a–d.

**
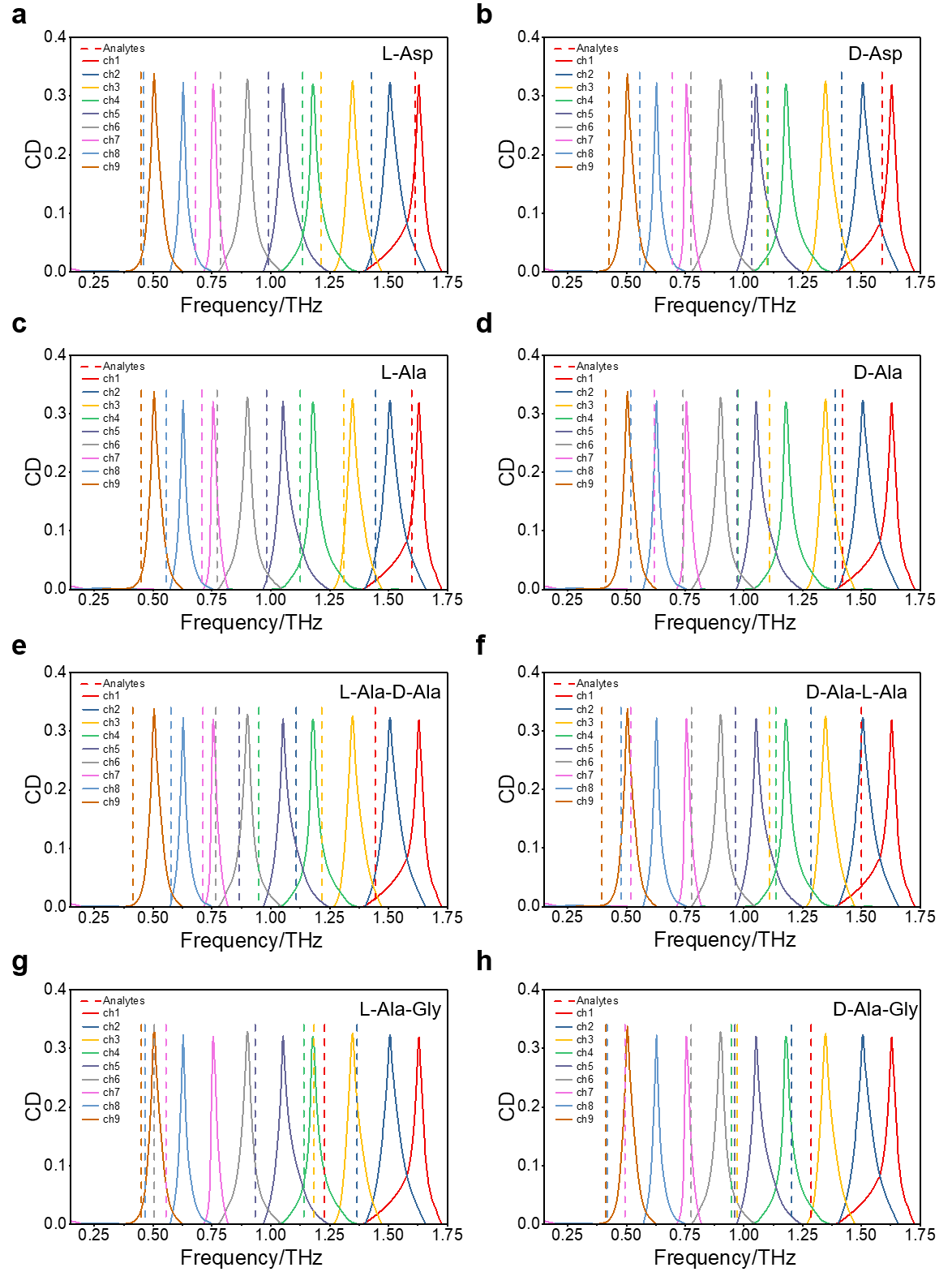
**

**Figure S11**: **Changes in multichannel terahertz CD after a mixture of another group of different chiral molecular solutions at a concentration of 0.05 mg/dL (C_1_). a** L-Aspartic. **b** D-Aspartic. **c** L-Alanine. **d** D-Alanine. **e** L- Alanine - D - Alanine. **f** D- Alanine - L - Alanine. **g** L-Ala-Gly .**h** D-Ala-Gly.

**
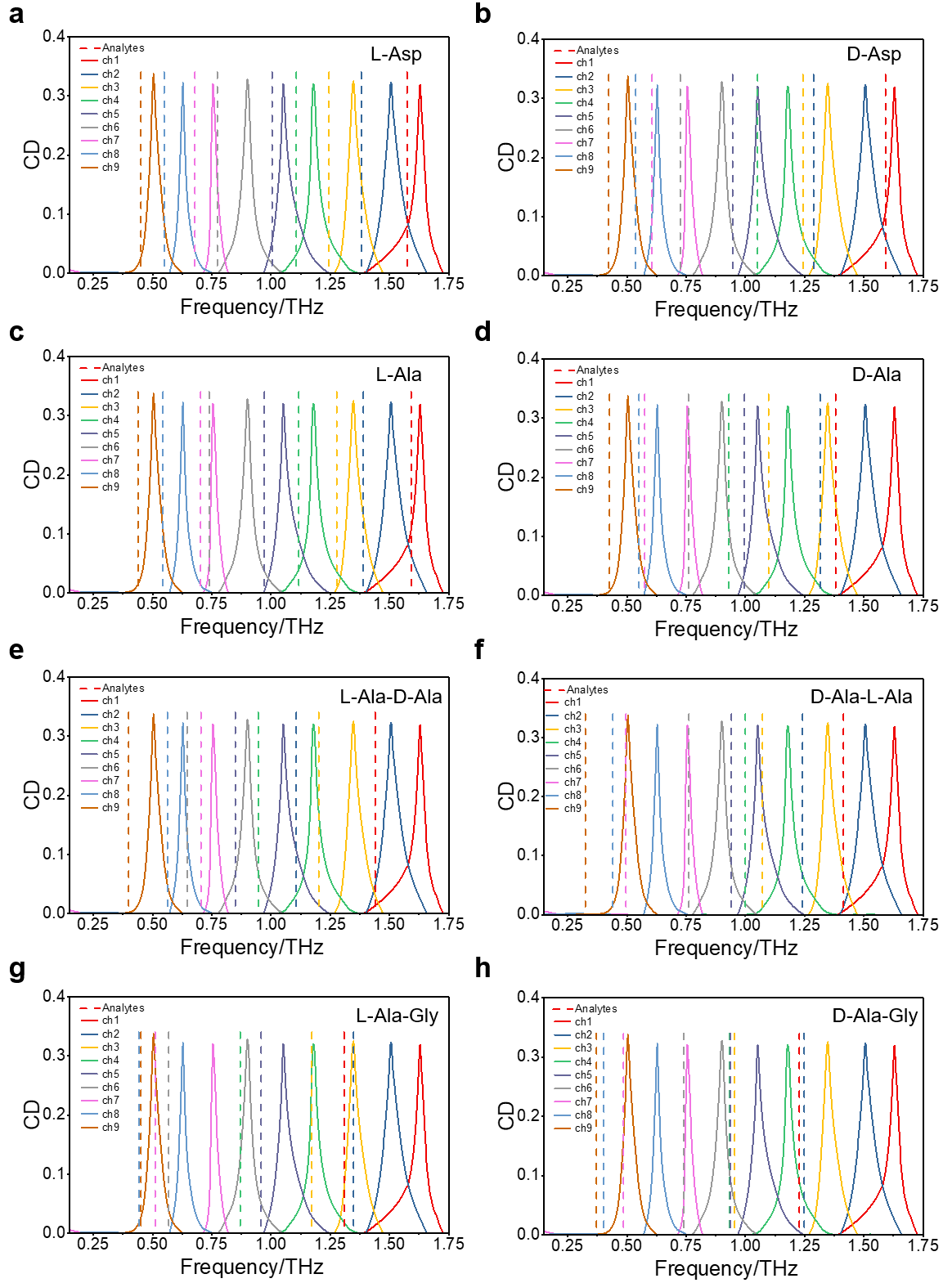
**

**Figure S12**: **Changes in multichannel terahertz CD after a mixture of another group of different chiral molecular solutions at a concentration of 0.15 mg/dL (C_2_). a** L-Aspartic. **b** D-Aspartic. **c** L-Alanine. **d** D-Alanine. **e** L- Alanine - D - Alanine. **f** D- Alanine - L - Alanine. **g** L-Ala-Gly .**h** D-Ala-Gly.

**
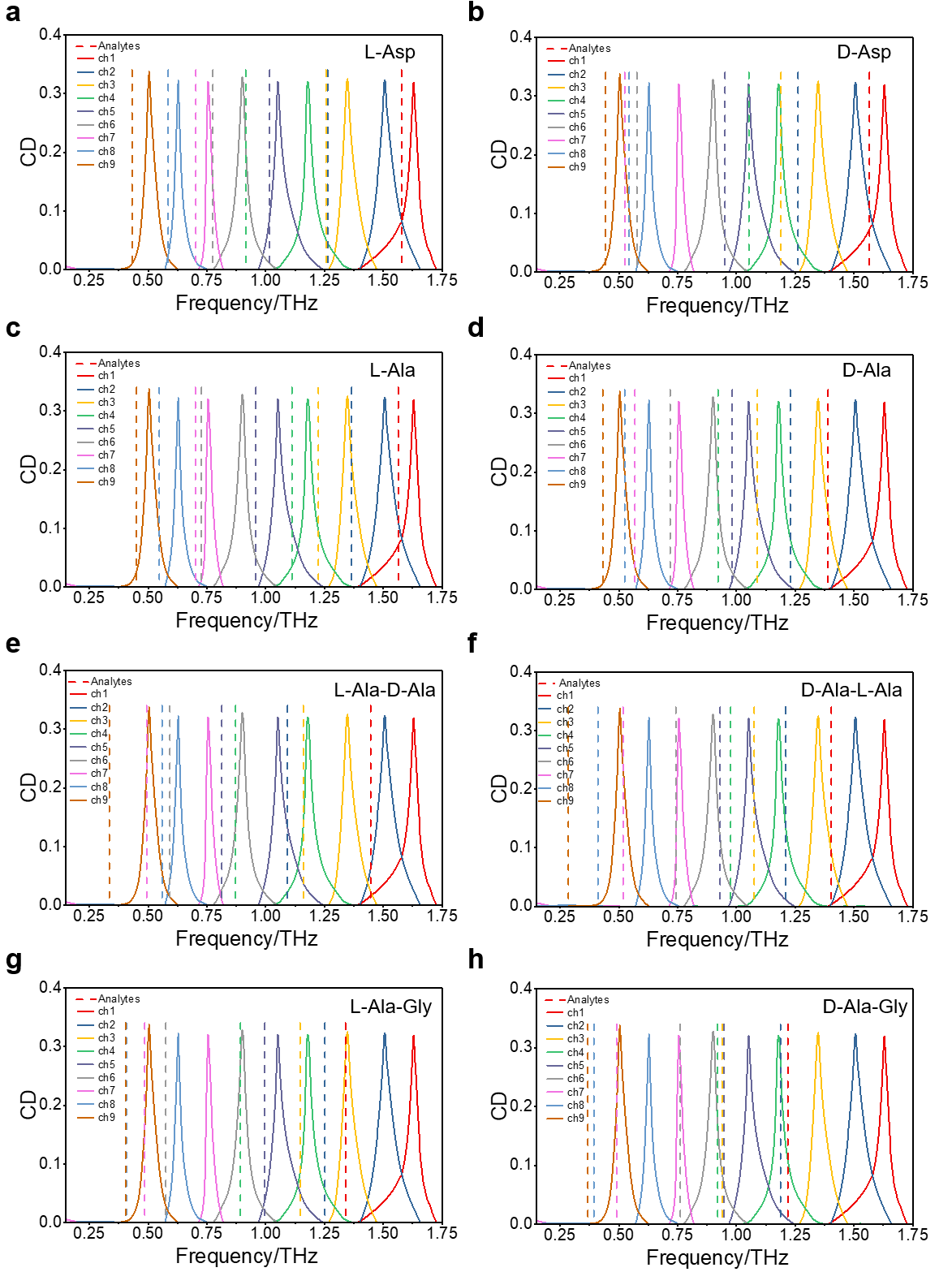
**

**Figure S13**: **Changes in multichannel terahertz CD after a mixture of another group of different chiral molecular solutions at a concentration of 0.3 mg/dL (C_3_). a** L-Aspartic. **b** D-Aspartic. **c** L-Alanine. **d** D-Alanine. **e** L- Alanine - D - Alanine. **f** D- Alanine - L - Alanine. **g** L-Ala-Gly .**h** D-Ala-Gly.
